# Neutrophils Contribute to Excess Serum BAFF Levels and Promote CD4^+^ T Cell and B Cell Responses in Lupus-Prone Mice

**DOI:** 10.1371/journal.pone.0102284

**Published:** 2014-07-10

**Authors:** Christine M. Coquery, Nekeithia S. Wade, William M. Loo, Jason M. Kinchen, Kelly M. Cox, Chao Jiang, Kenneth S. Tung, Loren D. Erickson

**Affiliations:** 1 Department of Microbiology, Immunology, and Cancer Biology, University of Virginia, Charlottesville, Virginia, United States of America; 2 Beirne B. Carter Center for Immunology Research, University of Virginia, Charlottesville, Virginia, United States of America; 3 Center for Cell Clearance, University of Virginia, Charlottesville, Virginia, United States of America; 4 Department of Pathology, University of Virginia, Charlottesville, Virginia, United States of America; 5 Division of Extramural Research, National Institutes of Health, Bethesda, Maryland, United States of America; New York University, United States of America

## Abstract

Despite increased frequencies of neutrophils found in autoimmune diseases such as systemic lupus erythematosus (SLE), how they contribute to disease pathogenesis and the mechanisms that affect the accumulation of neutrophils are poorly understood. The aim of this study was to identify factors in autoantibody-mediated autoimmunity that controls the accumulation of spleen resident neutrophils and to determine whether neutrophils contribute to abnormal B cell responses. Increased levels of the cytokine BAFF have been linked to loss of B cell tolerance in autoimmunity, but the cellular source responsible for excess BAFF is unknown. B cell maturation antigen (BCMA) is a receptor for BAFF and is critical for the survival of bone marrow plasma cells. Paradoxically, BCMA deficiency exacerbates the formation of autoantibody-secreting plasma cells in spleens of lupus-prone mice and the reasons for this effect are not understood. Here we analyzed the phenotype, localization and function of neutrophils in spleens of healthy mice and congenic lupus-prone mice, and compared mice sufficient or deficient in BCMA expression. Neutrophils were found to be significantly increased in frequency and activation status in spleens of lupus-prone mice when BCMA was absent. Furthermore, neutrophils localized within T cell zones and enhanced CD4^+^ T cell proliferation and IFNγ production through the production of BAFF. Reduced BAFF and IFNγ serum levels, decreased frequencies of IFNγ-producing T cells, germinal center B cells, and autoantibody production after neutrophil depletion indicated the involvement of neutrophils in these autoimmune traits. Thus, we have identified a novel role for BCMA to control excess BAFF production in murine lupus through restraining the accumulation of BAFF-producing neutrophils. Our data suggests that devising therapeutic strategies to reduce neutrophils in autoimmunity may decrease BAFF levels and ameliorate disease.

## Introduction

SLE is an autoimmune disorder characterized by a breakdown in B cell tolerance, leading to the generation of autoreactive plasma cells (PCs) that produce pathogenic autoantibodies. The factors that control the abnormal generation and maintenance of autoreactive PCs are poorly understood. Family members belonging to the B cell activating factor of the TNF family (BAFF) cytokine-receptor network have been closely linked to B cell homeostasis and tolerance [1], [2], [3]. B cell maturation antigen (BCMA) is a receptor expressed on PCs (but not on mature B cells) and is critical for survival of long-lived PCs in the bone marrow [4]. Signaling through BCMA on bone marrow PCs induces the expression of the anti-apoptotic molecule Mcl-1 that is necessary for survival [5]. In contrast, the BAFF receptor BR3 is expressed on mature B cells (but not on PCs) and is critical for their survival in peripheral tissues [6]. BR3 is also expressed on some CD4^+^ T cells and stimulates proliferation in response to BAFF [7], [8], [9], [10].

Excess circulating BAFF levels in both lupus-prone mice and SLE patients are associated with a loss of B cell tolerance and autoantibody production [11], [12], [13]. In lupus-prone mice, neutralizing BAFF activity reduces both the frequency of peripheral B cells and activation of T cells, which is sufficient to prevent and treat the disease [14], [15]. Yet, the mechanisms that control excess BAFF production in autoimmunity and which BAFF-producing cells contribute to disease pathogenesis are unknown.

The innate and adaptive arms of the immune system are thought to play essential roles in the development of SLE [16]. Neutrophils are a critical component of the innate immune system and the first line of defense against invading pathogens through uptake and destruction of microorganisms. The contribution of neutrophils to SLE pathology has been largely attributed to their ability to produce type I IFNs [16]. In addition, neutrophils undergo cell death by releasing neutrophil extracellular traps (NETs) that provide a source of autoantigens [17], [18], [19], [20]. Neutrophils produce BAFF that is stored intracellular as preformed molecules, which are released when cells are stimulated with IFNγ [21]. Neutrophils also express a membrane-anchored form of BAFF that is cleaved to a biologically active soluble form after stimulation [22]. Recently, a subset of human neutrophils has been shown to provide help to splenic B cells through the production of BAFF that enhances antibody production [23]. Thus, neutrophils may be a key cellular source of BAFF in SLE that contribute to abnormal B cell responses.

Given the important role of BCMA in maintaining long-lived PCs, we hypothesized that lupus-prone mice deficient in BCMA would have reduced survival of autoreactive PCs and therefore diminished pathogenic autoantibodies. Paradoxically, loss of BCMA in two different lupus-prone mouse models exacerbated disease through a CD4^+^ T cell-dependent mechanism that resulted in increased serum BAFF levels and autoantibody production despite reduced survival of bone marrow PCs [14]. Where and how BAFF production is controlled in these murine lupus models is unknown. We report that BCMA plays an important role in controlling the production of BAFF in autoimmunity. We found that BCMA deficiency resulted in an increased frequency of activated BAFF-producing neutrophils in spleens of lupus-prone mice. Interestingly, splenic neutrophils co-localized with CD4^+^ T cells and the accumulation of splenic neutrophils correlated with increased serum IFNγ titers and the frequency of IFNγ-producing T cells. Neutrophils from BCMA-deficient lupus-prone mice cultured with wild-type CD4^+^ T cells significantly promoted T cell proliferation and the production of IFNγ compared to neutrophils from control mice. These cellular responses were dependent on BAFF signaling through the BAFF receptor BR3 expressed on CD4^+^ T cells. Long-term depletion of neutrophils using the mAb, 1A8, significantly reduced the frequency of IFNγ-producing CD4^+^ T cells and the autoimmune phenotype in BCMA-deficient lupus-prone animals. These findings extend the role of neutrophils in the pathogenesis of lupus and suggest neutrophils help shape CD4^+^ T cell responses via BAFF that contribute to the production of pathogenic autoantibodies.

## Materials and Methods

### Mice

*Tnfrsf17^−/−^*, B6.Fas^lpr^/J, B6.Fas^lpr^/J*Tnfrsf17^−/−^*, *Nba2*, and *Nba2Tnfrsf17^−/−^* mice, fully backcrossed onto the C57BL/6 (B6) strain, were previously described [14], [24]. Age-matched WT B6 mice were purchased from NCI. Mice were screened by PCR to determine inheritance of the entire *Nba2* locus, the loss of *Tnfrsf17*, and *lpr* mutation as previously described [14]. All experiments were performed on individual mice using four-to-six month-old female mice unless indicated. Mice were randomized to experimental conditions and no mice were excluded from data analysis. For *in* *vivo* experiments, mice were treated in their home cage between the hours of 2–5 pm. Intra-peritoneal (I.P.) administration of 1A8 mAb was selected to achieve systemic responses.

### Ethics statement

Mice were housed in a specific pathogen-free animal facility at the University of Virginia. All animal procedures were conducted in compliance with the National Institutes of Health guidelines and were approved by the Institutional Animal Care and Use Committee of the University of Virginia (Protocol #3506). Animals received food and water *ad libitum* and were housed in groups of 5, whenever possible. All efforts were made to minimize suffering and mice were euthanized by carbon dioxide inhalation.

### *In vivo* pristane treatment and neutrophil depletion

Three-month-old female mice were given a single I.P. injection of 0.5 ml PBS (Gibco) or TMPD (2,6,10,14-tetramethylpentadecane), commonly known as pristane (Sigma-Aldrich), as previously described [25]. After one month, animals were euthanized and spleen and serum were prepared for flow cytometric analysis of neutrophils, T- and B-cell populations, and ELISA to measure serum BAFF, IFNγ, and autoantibody titers. To deplete neutrophils, B6.Fas^lpr^/J*Tnfrsf17* ^−/−^ mice were given I.P. injections of 400 µg rat IgG (Sigma-Aldrich) or 1A8 (BioXCell) every other day for 4 weeks. Following treatment, mice were euthanized and spleen and serum were harvested for analysis.

### *In vitro* assays

CD4^+^ T cells from spleens of WT mice were isolated to greater than 90% purity (Miltenyi MACS kit). Neutrophils from spleens of lupus-prone mice were isolated to greater than 90% purity, as previously described [26]. All cells were cultured in complete RPMI (Gibco) supplemented with 10% FBS (Gibco) unless otherwise indicated. Cell viability was determined using live/dead AQUA (Invitrogen) according to manufacturer’s instructions. For T cell and neutrophil co-cultures, CD4^+^ T cells were labeled with CellTrace Violet (Life Technologies) and cultured (5×10^4^) alone or with equivalent numbers of splenic neutrophils in the presence of 1 µg/ml anti-CD3 (145-2C11, CedarLane Labs) for 3 days. In some cases, 10 µg/ml of anti-BR3 blocking antibody was added (R&D Systems). T cells were analyzed by flow cytometry for proliferation by CellTrace Violet dilution and culture supernatants were analyzed for IFNγ, IL-4, and IL-17 production by ELISA. To measure the frequency of IFNγ-producing CD4^+^ T cells, spleen cells were cultured in the presence of 50 ng/ml PMA (Sigma-Aldrich), 500 ng/ml ionomycin (Sigma-Aldrich) in the presence of GolgiStop (BD Biosciences). After 5 hours of stimulation, CD4^+^ T cells that produced IFNγ were measured by intracellular flow staining.

### Flow cytometry

Mouse spleen single-cell suspensions were prepared as previously described [26]. Cells were spun at 800 rpm (130 G) for 8 minutes and maintained on ice for all manipulations. Cells were stained at a concentration of 1−2×10^6^ cells/well in a 100 µl volume. Following staining, cells were fixed in 1% formaldehyde (Fisher) or Fix/Perm Buffer (eBioscience). Mouse antibodies included the following: Ly6G-PE or Ly6G-PECy7 (1A8), CD16/CD32-Alexa647 (93), CD64-PE (X54-5/7.1), CD62L-PE or CD62L-APC (MEL-14), CD11a-FITC (M17/4), GR-1-FITC (RB6-8C5), all from BioLegend; CD11b-FITC or CD11b-PE (M1/70), CD18-biotin (M18/2), CD4-EF450 (GK1.5), all from eBioscience; IFNγ-PE (XMG1.2), B220-PE (RA3-6B2), CD138-APC (281-2), GL-7-FITC, all from BD Biosciences. Streptavidin was used on the following fluorophores: FITC, PE, APC, EF450, APC-Cy7. Viability was determined using LIVE/DEAD Fixable AQUA (Life Technologies). Samples were acquired on a CyAn ADP (Beckman Coulter) and analyzed using FlowJo software version 9.3.3 (TreeStar Inc.). Gates were first set on live cells, singlets, and FMO (fluorescence minus one) controls.

### Imagestream

To confirm the morphology of neutrophils, cells were stained with anti-CD11b-FITC and anti-Ly6G-PE-Cy7 as described above. Neutrophils were fixed and permeabilized using the BD Cytofix/Cytoperm Kit according to manufacturer’s instructions (Becton Dickinson). DAPI (Life Technologies) was used for nuclear staining. Samples were acquired on an ImageStreamX (Amnis) at a 60X magnification and analyzed using IDEAS software.

### Histology and immunofluorescence

Frozen 5 µm tissue sections were stained with the following reagents: PNA-FITC (Sigma-Aldrich); IgD-EF450 (11–26) and CD11b-PE (M1/70) from eBioscience; Ly6G-APC (1A8), CD11c-biotin (N418) from BioLegend; BAFF-PE (121808) from BD Bioscience; C3-FITC (CL7503F) from CedarLane Labs; IgA-biotin or IgG-biotin from Southern Biotech. Streptavidin-EF450 was purchased from eBioscience. Sections were analyzed on an Axio Imager 2 with Apotome (Zeiss). Magnification is provided in the figure legend. Quantification of BAFF intensity in sections was performed using the open source software CellProfiler (Broad Institute) and is given in arbitrary units (A.U.). H&E staining was performed as previously described [14].

### ELISAs

Murine BAFF (Apotech) and IFNγ (R&D) levels in culture supernatant or sera were measured by ELISA. IL-4 and IL-17 levels in culture supernatant were measured using the following reagents from eBioscience: anti-mouse IL-4 (11B11), anti-mouse IL-17 (MM17F3), anti-mouse IL-4 biotin (BVD6-24G2), anti-mouse IL-17 biotin (17B7). Recombinant murine IL-4 and IL-17 were used to generate a standard curve. Serum autoantibody titers were measured as previously described [14].

### Real-time PCR

Cells were suspended in RLT/2-ME buffer and total RNA was isolated using the RNAqueous Micro Kit (Ambion). cDNA was generated using the iScript cDNA synthesis kit (Bio-Rad), according to manufacturer’s instructions. PCR reactions were performed using iQSYBR Green Supermix (Bio-Rad) and the following primer sequences (IDT Technologies): *HPRT* Fwd-5′-AGC TAC TGT AAT GAT CAG TCA ACG-3′, *HPRT* Rev-5′-AGA GGT CCT TTT CAC CAG CA-3′, *BCMA* Fwd-5′-GGC GCA ACA GTG TTT CCA CA-3′, *BCMA* Rev-5′-CTC GGT GTC GGC CTT GTC CA-3′. Samples were run on a BioRad MyiQ System.

### Statistical analysis

Statistics were determined using Prism software v5.0 (GraphPad Software). All error bars represent the mean ± standard error of the mean. All p-values were derived from the two-tailed Student’s t test, one-way ANOVA, or two-way ANOVA, as appropriate, and stated in the figure legend (*p<0.05, **p<0.01, ***p<0.001).

## Results

### BAFF levels are influenced by BCMA in murine lupus

**Figure 1:**
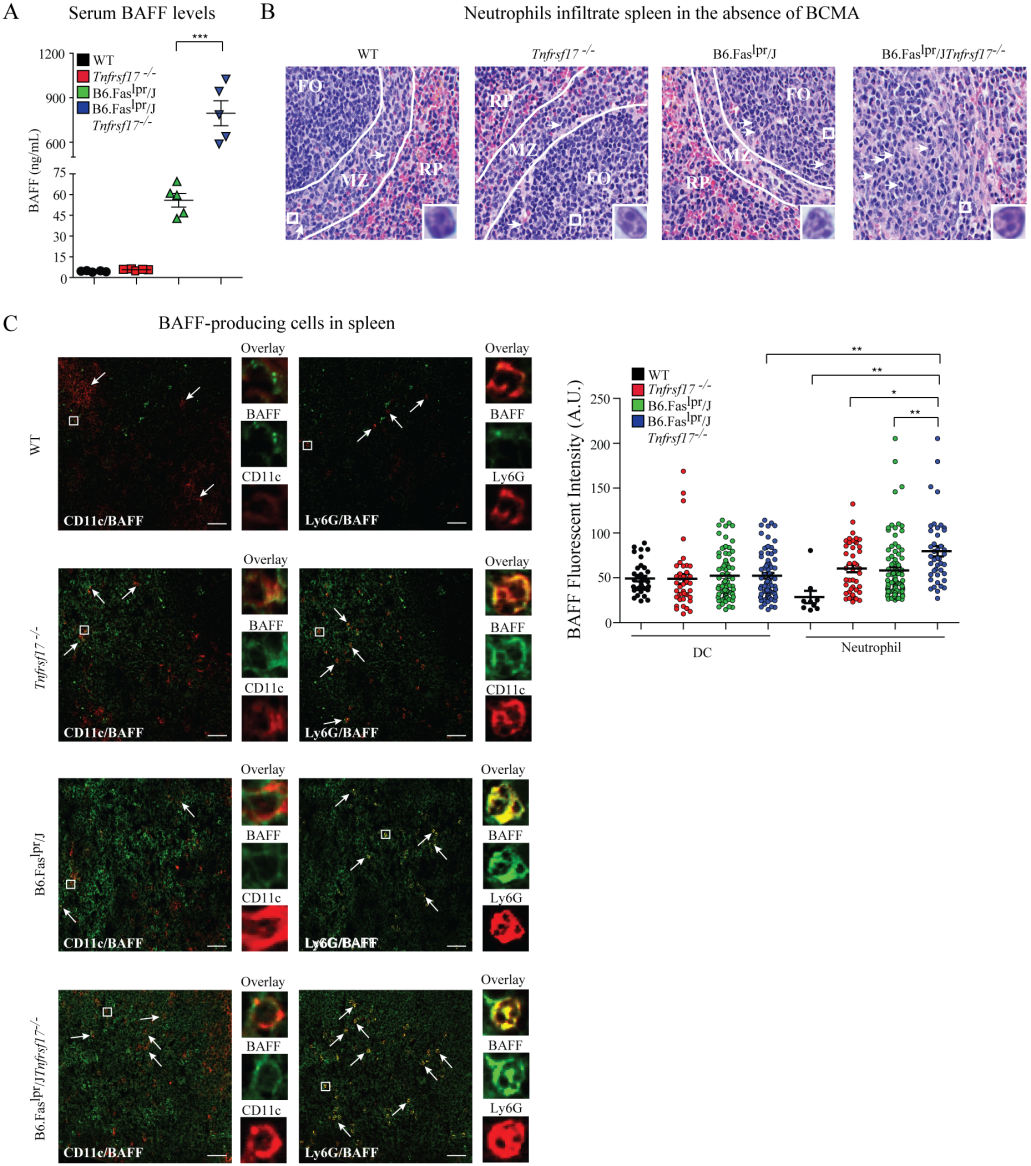
Neutrophils and dendritic cells contribute to elevated BAFF levels in the absence of BCMA. (A) BAFF levels were measured in sera from mice of the indicated genotype. Each symbol represents a single animal. (B) Spleen sections from 6 month-old mice were stained with H&E. Each arrow represents a neutrophil identified by nuclear structure, with the boxed neutrophil magnified 100x in the inset image. Shown are representative images from 5 mice per genotype. RP – red pulp, FO – follicle, MZ – mantle zone. (C) Left panels: spleen sections from 6-month-old mice were analyzed for BAFF-producing cells by immunofluorescence. BAFF – green, CD11c or Ly6G – red. Scale bar represents 50 µm. Arrows represent cells with co-localization of BAFF and either CD11c or Ly6G, with the boxed cell magnified in the inset images. One representative image from 4 mice of each genotype is shown. Right panel: quantification of the intensity of BAFF staining from spleen-resident CD11c^+^ DCs and Ly6G^+^ neutrophils was measured by CellProfiler. Each symbol represents an individual cell within a single histological sample from a minimum of 3 distinct samples/genotype. Error bars indicate mean ± SEM. Statistics determined using a one-way ANOVA with a Tukey post test (A, C), and denoted as follows: *p<0.05, **p<0.01, ***p<0.001.

We previously demonstrated that lupus-prone mice with a deficiency in BCMA leads to increased serum BAFF levels [14]. To evaluate further the role of BCMA on the expression of BAFF in autoimmunity, we studied the BAFF-producing innate immune cells in spleens of female naïve C57BL/6 (WT) mice and congenic lupus-prone B6.Fas^lpr^/J mice, and compared mice sufficient or deficient in the gene *Tnfrsf17* that encodes BCMA. Twenty-five-week old mice were analyzed due to robust differences in the amount of circulating BAFF among the genotypes. B6.Fas^lpr^/J and B6.Fas^lpr^/J*Tnfrsf17^−/−^* mice showed increased serum BAFF levels, with significantly higher BAFF levels in the absence of BCMA compared to WT and *Tnfrsf17^−/−^*controls, indicating that heightened BAFF levels were dependent on *lpr* predisposition (Fig. 1A). These results confirm our previous findings, which further demonstrated no difference in APRIL expression among the mouse strains [14]. We previously showed increased frequencies of splenic CD11c^+^ dendritic cells (DC) in B6.Fas^lpr^/J*Tnfrsf17^−/−^* mice compared to B6.Fas^lpr^/J mice [14], suggesting that the heightened level of circulating BAFF in these mice may be derived from elevated numbers of BAFF-producing DCs. Interestingly, hematoxylin and eosin staining of spleen sections revealed significant accumulation of polymorphonuclear (PMN) leukocytes with a neutrophil phenotype in B6.Fas^lpr^/J*Tnfrsf17^−/−^* mice compared to the other strains (Fig. 1B). PMNs in the spleens of B6.Fas^lpr^/J*Tnfrsf17^−/−^* mice were found in abundance surrounding enlarged follicles with a poorly defined mantle zone, whereas fewer PMNs surrounding follicles were observed in spleens of B6.Fas^lpr^/J mice. Analysis of spleens from WT and *Tnfrsf17^−/−^* mice indicated that PMNs were found predominantly within the mantle zone. These data suggest that neutrophils, together with DCs, may contribute to excess BAFF production in autoimmunity and are modulated in cell frequency or BAFF production by BCMA.

To determine whether BCMA deficiency affects the accumulation and BAFF expression of DCs and neutrophils, we examined these cells by immunofluorescent staining for Ly6G and CD11c and quantified BAFF co-localization in spleens of mice. Confocal imaging confirmed increased frequencies of CD11c^+^ DCs in spleens of B6.Fas^lpr^/J*Tnfrsf17^−/−^* mice compared to the other strains (Fig. 1C, left panel). Despite increased splenic CD11c^+^ DCs in B6.Fas^lpr^/J*Tnfrsf17^−/−^* mice, we observed no differences in CD11c^+^ cells that co-localized with BAFF expression compared to the other strains. To verify this observation, we quantified the intensity of fluorescently stained BAFF on each CD11c^+^ cell as a measurement of the amount of BAFF protein using the image analysis software, CellProfiler [27]. No significant differences in BAFF intensities co-localizing to CD11c^+^ DCs were measured among the mouse strains (Fig. 1C, graph). We also did not observe any differences in BAFF intensities co-localizing to F4/80^+^ cells, suggesting that BCMA deficiency does not affect BAFF protein expression in splenic macrophages (data not shown).

Neutrophils are phenotypically defined as Ly6G^+^ CD11b^+^ and have been shown to be potent BAFF producers [23], [28], [29]. To investigate further whether BCMA deficiency controls BAFF production via neutrophils, we examined Ly6G and BAFF expression in spleen. WT and *Tnfrsf17^−/−^* mice had few Ly6G^+^ cells present in spleen (Fig. 1C, right panel). In contrast, B6.Fas^lpr^/J and B6.Fas^lpr^/J*Tnfrsf17^−/−^* mice consistently had higher frequencies of splenic Ly6G^+^ cells. Quantification of BAFF intensity per Ly6G^+^ cell demonstrated increased BAFF intensities of Ly6G^+^ cells in spleens of *Tnfrsf17^−/−^* and B6.Fas^lpr^/J mice, with higher BAFF intensities in B6.Fas^lpr^/J*Tnfrsf17^−/−^* mice compared to WT controls (Fig. 1C). Taken together, these data indicate that BCMA deficiency in lupus-prone mice controls the abnormal accumulation of splenic CD11c^+^ DCs and neutrophils, and suggests that neutrophils contribute to excess circulating BAFF levels in B6.Fas^lpr^/J*Tnfrsf17^−/−^* mice.

### BCMA deficiency in lupus-prone mice promotes accumulation of spleen-resident neutrophils

**Figure 2:**
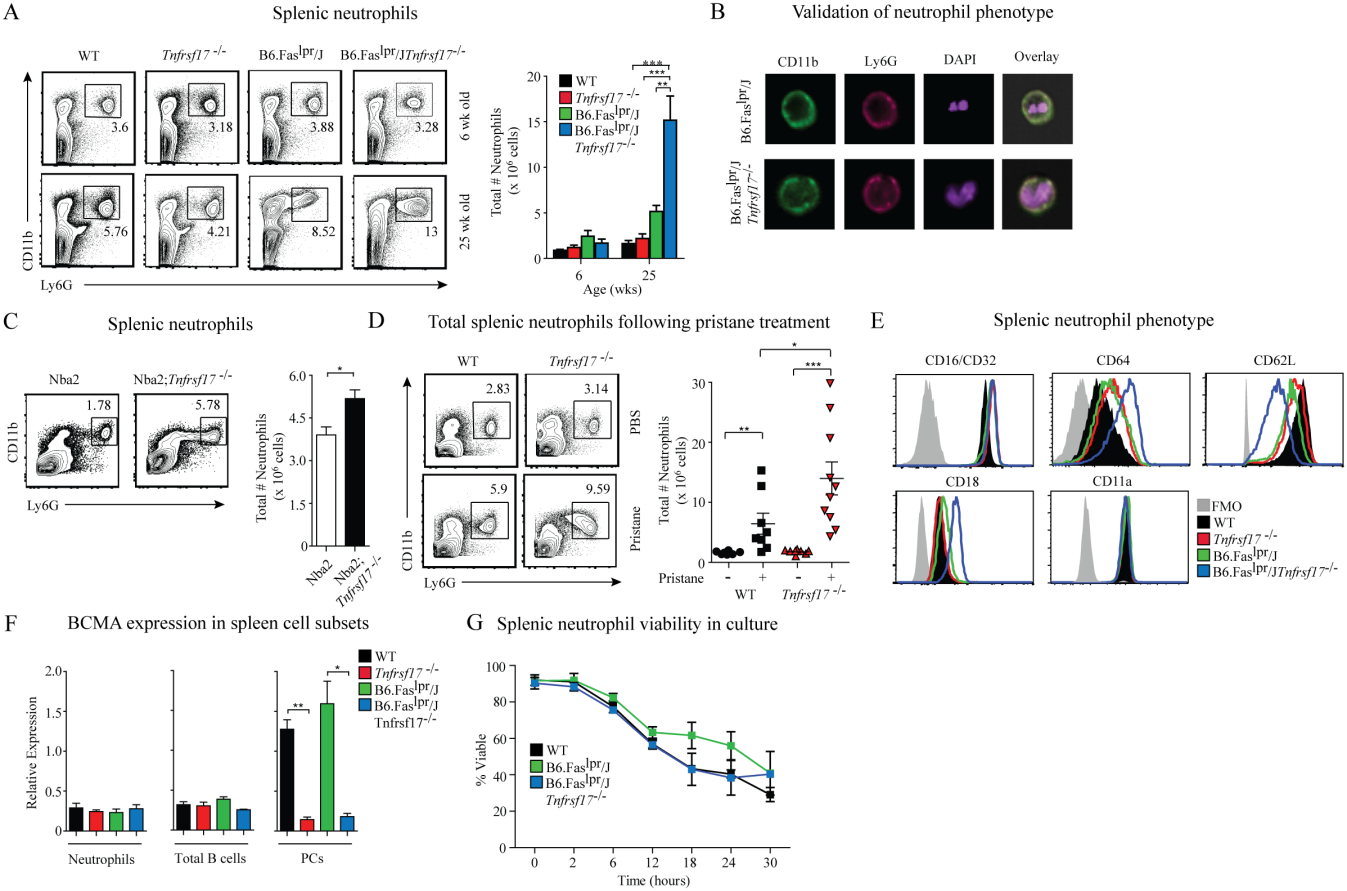
Neutrophils accumulate and have an activated phenotype in spleens of lupus-prone in the absence of BCMA. (A) The frequency and total number of splenic neutrophils (CD11b^+^Ly6G^+^) was determined in mice of the indicated genotype at 6 and 25 weeks of age. Left panel: representative flow cytometry plots showing the percentages of neutrophils for each strain. Right panel: total numbers of neutrophils in spleens of mice were quantified from 6–9 mice/genotype. (B) Neutrophil nuclear morphology was validated using ImageStream technology by measuring co-localization of CD11b, Ly6G and the presence of a multi-lobed nucleus using DAPI. One representative image/genotype from at least 100 images/genotype is shown. Images were taken at 60X. (C) The frequency of neutrophils in spleens of *Nba2* and *Nba2;Tnfrsf17^−/−^* mice were quantified from 9 mice per strain. (D) Three-month-old WT and *Tnfrsf17^−/−^* mice were injected with a single dose of either PBS or pristane. After 4 weeks, the frequency of neutrophils in spleens of mice was determined. Left panel: representative flow cytometry plots showing the percentages of neutrophils for each group. Right panel: total numbers of neutrophils for each group; each symbol represents an individual animal. Combined data from two independent experiments. (E) Representative histograms showing the activation state of neutrophils freshly isolated from spleens of mice compared to FMO (solid grey) and WT (solid black) controls. One histogram from 5 individual mice/genotype analyzed. (F) Gene expression values of *Tnfrsf17* relative to *HPRT* in sorted cells from spleens of mice were determined by qPCR on total RNA. Combined data from three independent mice/genotype. (G) The percent of viable neutrophils was measured at the indicated time-points from purified splenic neutrophils using LIVE/DEAD Fixable AQUA. Combined data from 3 mice/genotype. Error bars indicate mean ± SEM. Statistics determined with a one-way ANOVA using a Tukey post test (A, D) or Student’s t test (C), and denoted as follows: *p<0.05, **p<0.01, ***p<0.001.

We next evaluated the steady state frequencies of splenic neutrophils in 6-week-old and 25-week-old mice. No differences in the percentages and numbers of Ly6G^+^CD11b^+^ neutrophils were observed among 6-week-old mice (Fig. 2A and data not shown). In contrast, B6.Fas^lpr^/J*Tnfrsf17^−/−^* mice showed significantly higher numbers of splenic neutrophils at 25 weeks of age compared to the other strains. ImageStream analysis confirmed that the Ly6G^+^CD11b^+^ cells isolated from spleens of B6.Faslpr/J and B6.Fas^lpr^/J*Tnfrsf17^−/−^* mice were neutrophils, as defined by the classic polymorphonuclear granulocyte morphology (DAPI staining; Fig. 2B). We did not observe any differences in the frequencies of Ly6G^+^CD11b^+^ neutrophils in the bone marrow of lupus-prone mice compared to control animals, suggesting that the increased frequency of splenic neutrophils in lupus-prone mice resulted from accumulation and not increased neutrophil development (data not shown). Increased frequencies of splenic neutrophils were also found in BCMA deficient congenic *Nba2* lupus-prone mice [14], [24], which provided independent confirmation that BCMA expression is critical for restraining accumulation of neutrophils in autoimmunity (Fig. 2C). Finally, to determine whether these findings were limited to murine autoimmune susceptibility genes, we examined splenic neutrophils in WT and *Tnfrsf17^−/−^* mice under environmentally induced lupus conditions after treatment with the naturally occurring hydrocarbon oil pristane. Administration of pristane to naïve wild-type mice results in chronic inflammation and increased production of IFNγ that induces a mild lupus-like disease, including loss of B cell tolerance [25]. *Tnfrsf17^−/−^* mice had significantly higher numbers of splenic neutrophils compared to WT mice following pristane treatment and mock-treated controls (Fig. 2D). These data indicate that BCMA is a key regulator of splenic neutrophil accumulation in autoimmunity.

A previous report has demonstrated that increased BAFF production by neutrophils is linked with an elevated activation state [21]. Following activation, neutrophils upregulate the low affinity IgG Fc receptors (CD16/CD32), the high affinity receptor for IgG (CD64), and LFA-1 (comprised of CD11a and CD18), as well as downregulate CD62L expression [30], [31], [32]. Therefore, we tested whether the increased frequency of splenic BAFF-producing neutrophils from B6.Fas^lpr^/J*Tnfrsf17^−/−^* mice was associated with cell activation using these markers. We observed minimal differences in membrane expression levels of CD11a and CD16/CD32 on neutrophils among the strains (Fig. 2E). In contrast, neutrophils from B6.Fas^lpr^/J*Tnfrsf17^−/−^* mice had markedly higher expression levels of CD64 and CD18, and lower CD62L compared to neutrophils from control mice. Splenic neutrophils from *Nba2*;*Tnfrsf17^−/−^* mice also expressed increased levels of CD64 and CD18, and decreased CD62L expression (data not shown). These data indicate that the absence of BCMA in lupus-prone mice contributes to an activated phenotype of spleen-resident neutrophils.

We have recently shown in lupus-prone mice that, in addition to plasma cells, BCMA is expressed in a subset of CD4^+^ T cells and serves as a negative regulator of the germinal center response (Coquery et al, manuscript submitted). To determine if BCMA is expressed in neutrophils and therefore directly involved in controlling the accumulation of splenic neutrophils in lupus-prone mice, we analyzed BCMA expression by measuring mRNA transcript levels. We chose this method since detection of BCMA protein on the cell surface of murine cells is unreliable due to poor reagents [33]. Using a protocol developed by our laboratory to isolate spleen-resident murine neutrophils [26], we found that BCMA was not expressed in neutrophils from B6.Fas^lpr^/J mice compared to WT, *Tnfrsf17^−/−^*, and B6.Fas^lpr^/J*Tnfrsf17^−/−^* mice (Fig. 2F). Plasma cells from WT and B6.Fas^lpr^/J mice served as a positive control, whereas naïve B cells that do not express BCMA served as negative controls. Additionally, no differences in the viability of purified neutrophils among the mouse strains were observed (Fig. 2G). These data indicate that the accumulation and activation of neutrophils in B6.Fas^lpr^/J*Tnfrsf17^−/−^* spleens is not due to BCMA deficiency in neutrophils, but results from the loss of BCMA in other cell types.

### Neutrophils co-localize with CD4^+^ T cells in spleens of lupus-prone mice and contribute to T cell response through a BAFF-dependent mechanism

**Figure 3:**
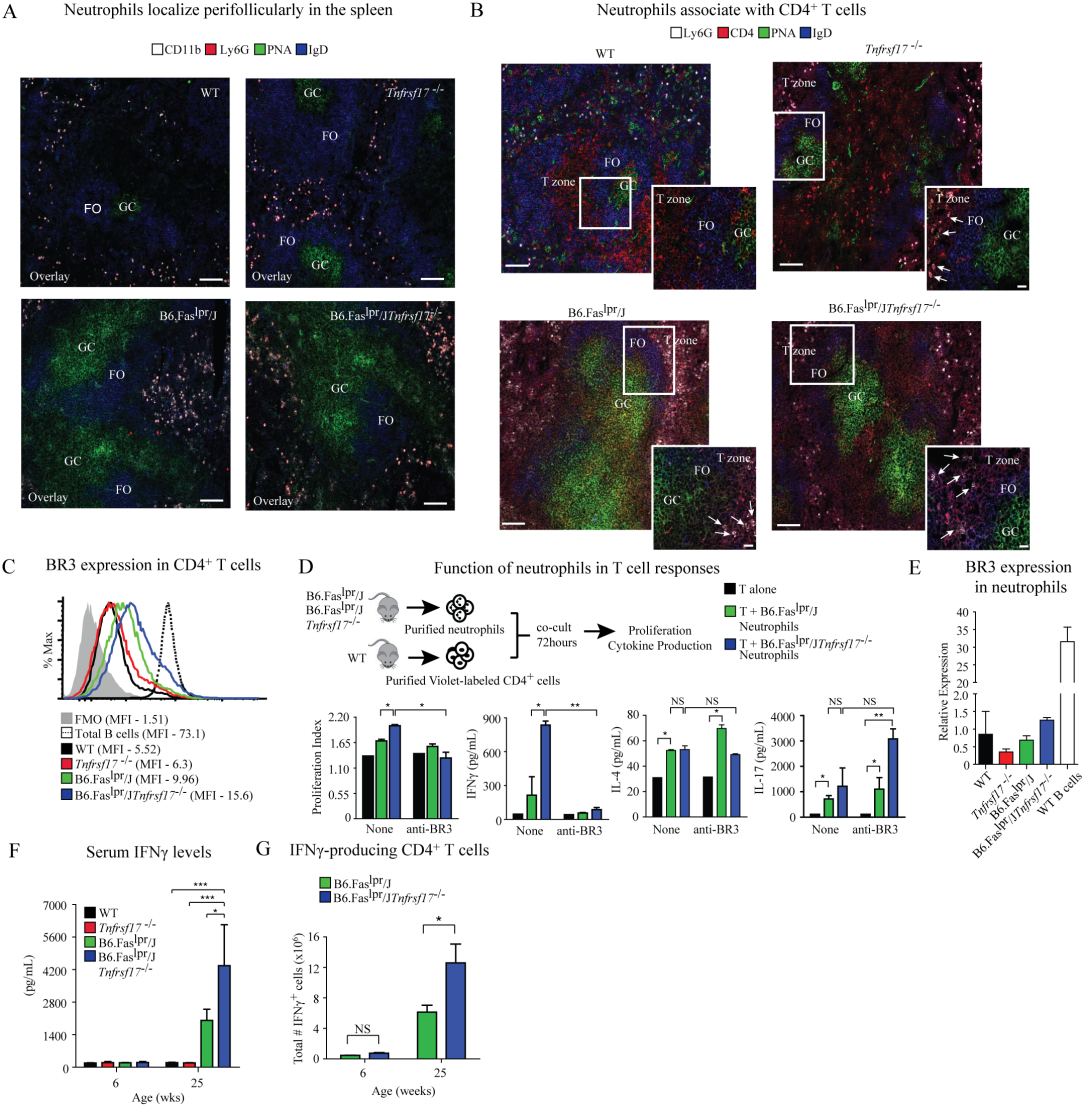
Neutrophils co-localize in T cell zones and influence CD4^+^ T cell responses in a BAFF-dependent manner. (A) Confocal microscopy of spleen sections demonstrating the *in* *situ* localization of neutrophils. CD11b – white, Ly6G – red, IgD – blue, PNA – green. Scale bar indicates 100 µm. (B) Confocal microscopy of spleen sections demonstrating the co-localization of neutrophils and CD4^+^ T cells. Ly6G – white, CD4– red, IgD – blue, PNA – green. Scale bar indicates 100 µm. Scale bar of inset indicates 20 µm. Images are representative of at least three images from 4 mice/genotype. (C) Representative histograms showing BR3 expression on CD4^+^ T cells from spleens of three mice/genotype. Total B cells (B220^+^) and a FMO stain served as controls. (D) Purified neutrophils from spleens of lupus-prone mice were co-cultured with CD4^+^ T cells from WT mice at a 1∶1 ratio in the presence or absence of BR3 blocking antibody. After 3 days of culture, CD4^+^ T cell proliferation was measured by CellTrace Violet dilution and cytokine concentrations in culture supernatant were measured by ELISA. Combined data from three mice/genotype. One of three independent experiments with similar results is shown. (E) Gene expression levels of *Tnfrsf13C* (BR3) in purified neutrophils from mice of the indicated genotype. Samples were normalized to *HPRT* and the expression in WT animals was set to one. Total B cells are shown as a positive control. Combined data from 3 mice/genotype. (F) Serum IFNγ levels from 6- and 25-week-old mice of the indicated genotype were measured by ELISA. Combined data from 5–9 mice/genotype. (G) Total splenocytes from mice of the indicated genotype were cultured in the presence or absence of PMA/ionomycin with GolgiStop. The frequency of IFNγ-producing CD4^+^ T cells was assessed following 5 hour *in* *vitro* stimulation. Combined data from 4 mice/genotype at 6 and 25weeks of age. Error bars indicate mean ± SEM. Statistics determined with a two-way ANOVA using a Bonferroni post test (D), or a one-way ANOVA with a Tukey post test (F, G), and denoted as follows: *p<0.05, **p<0.01, ***p<0.001, ns – not significant.

Low numbers of BAFF-producing neutrophils have been detected in the perifollicular regions of spleens from healthy humans [23]. To determine further where neutrophils accumulate in spleens of lupus-prone mice under steady state conditions, we stained tissue sections for Ly6G and CD11b, as well as for PNA and IgD to identify B cell follicles (PNA^−^IgD^+^) and germinal centers (PNA^+^IgD^−^). We detected Ly6G^+^CD11b^+^ neutrophils in the perifollicular area of spleens from WT mice (Fig. 3A). Neutrophils were also detected outside B cell follicles and germinal centers in spleens of *Tnfrsf17^−/−^* and B6.Fas^lpr^/J mice, with even more perifollicular neutrophils that extended outward, beyond the follicular mantle in spleens of B6.Fas^lpr^/J*Tnfrsf17^−/−^* mice. Interestingly, neutrophils were positioned in the T cell zones of *Tnfrsf17^−/−^*, B6.Fas^lpr^/J and B6.Fas^lpr^/J*Tnfrsf17^−/−^* mice compared to WT mice, as measured by co-localization of Ly6^+^ and CD4^+^ cells (Fig. 3B). These data suggest that splenic neutrophils accumulating in the T cell zones of B6.Fas^lpr^/J mice, and even more in B6.Fas^lpr^/J*Tnfrsf17^−/−^* mice, may interact with CD4^+^ T cells.

We hypothesized that the accumulation of splenic neutrophils in T cell zones of lupus-prone mice may influence CD4^+^ T cell responses through a BAFF-dependent mechanism. A portion of murine splenic mature CD4^+^ T cells express low levels of the BAFF receptor BR3 [8], [34], [35]. Consistent with these studies, we found that BR3 is expressed at low levels on splenic CD4^+^ T cells of all mouse strains compared to the high expression levels of BR3 on B cells (Fig. 3C). BR3 expression levels on CD4^+^ T cells from lupus-prone mice were slightly higher, as measured by mean fluorescence intensity (MFI) values but were not significant. To directly test whether splenic neutrophils from lupus-prone mice affected CD4^+^ T cell function, we developed a co-culture system to measure neutrophil-mediated CD4^+^ T cell responses *in* *vitro*. Purified neutrophils from B6.Fas^lpr^/J and B6.Fas^lpr^/J*Tnfrsf17^−/−^* mice were co-cultured with mature resting CD4^+^ T cells from WT mice and T cell proliferation and cytokine production in the culture supernatants were measured. Using this assay, we measured greater CD4^+^ T cell proliferation from co-cultures with neutrophils from B6.Fas^lpr^/J*Tnfrsf17^−/−^* mice compared to B6.Fas^lpr^/J mice, and T cells cultured alone (Fig. 3D). Confirmation that neutrophils from B6.Fas^lpr^/J*Tnfrsf17^−/−^* mice induced T cell proliferation by producing BAFF that signals through BR3 on CD4^+^ T cells was determined by adding a BR3 neutralizing mAb to the cultures, which reduced T cell proliferation (Fig. 3D). Neutrophils do not express BR3 and therefore cannot respond to BAFF (Fig. 3E). These data are consistent with our earlier findings that neutrophils from B6.Fas^lpr^/J*Tnfrsf17^−/−^* mice have a more robust activated phenotype compared to neutrophils from B6.Fas^lpr^/J mice (Fig. 2D).

Previous work using an OT-II system suggests that neutrophils may induce both Th1 and Th17 differentiation [36]. To determine whether autoimmune-prone neutrophils are driving different effector cell subsets, we co-cultured resting WT CD4^+^ T cells with neutrophils from B6.Fas^lpr^/J or B6.Fas^lpr^/J*Tnfrsf17^−/−^* mice and measured IFNγ, IL-4 and IL-17 in culture supernatant to determine the relative differentiation of Th1, Th2, and Th17 responses, respectively. The results demonstrated that T cells cultured alone produced low amounts of IFNγ, IL-4 and IL-17 (Fig. 3D). Addition of neutrophils from both B6.Fas^lpr^/J and B6.Fas^lpr^/J*Tnfrsf17^−/−^* mice to the cultures increased IFNγ production by T cells, with the IFNγ levels induced by neutrophils from B6.Fas^lpr^/J*Tnfrsf17^−/−^* mice substantially higher. Neutrophils from both B6.Fas^lpr^/J and B6.Fas^lpr^/J*Tnfrsf17^−/−^* mice promoted minimal IL-4 responses and induced equivalent levels of IL-17 (Fig. 3D). To evaluate the relative contribution of BAFF generated by neutrophils from lupus-prone mice to mediate cytokine production in CD4^+^ T cells of WT mice, we utilized a BR3 blocking mAb. Blocking BR3 reduced IFNγ production by T cells to basal levels, indicating that BAFF produced by splenic neutrophils is responsible for IFNγ production in CD4^+^ T cells in this system. However, blocking BR3 had no impact on IL-4 production. Interestingly, blocking BR3 increased the IL-17 production by T cells when cultured with neutrophils from B6.Fas^lpr^/J*Tnfrsf17^−/−^* mice but not B6.Fas^lpr^/J mice; however this increase was not statistically significant (Fig. 3D).

Excess production of IFNγ by CD4^+^ T cells is associated with the pathogenesis of SLE [37]. Analysis of serum IFNγ titers in lupus-prone mice demonstrated significantly higher levels from B6.Fas^lpr^/J*Tnfrsf17^−/−^* mice with age compared to B6.Fas^lpr^/J mice and control animals, which had little IFNγ (Fig. 3F). Moreover, increased numbers of IFNγ-producing CD4^+^ T cells in spleens of B6.Fas^lpr^/J*Tnfrsf17^−/−^* mice were observed compared to B6.Fas^lpr^/J mice (Fig. 3G). These data suggest that the elevated amount of BAFF generated by neutrophils in B6.Fas^lpr^/J*Tnfrsf17^−/−^* mice compared to B6.Fas^lpr^/J mice signals through BR3 on CD4^+^ T cells to preferentially drive the differentiation of Th1 cells.

### Depletion of neutrophils reduces excess BAFF and IFNγ levels and autoimmunity in B6.Fas^lpr^/J*Tnfrsf17^−/−^* mice

**Figure 4:**
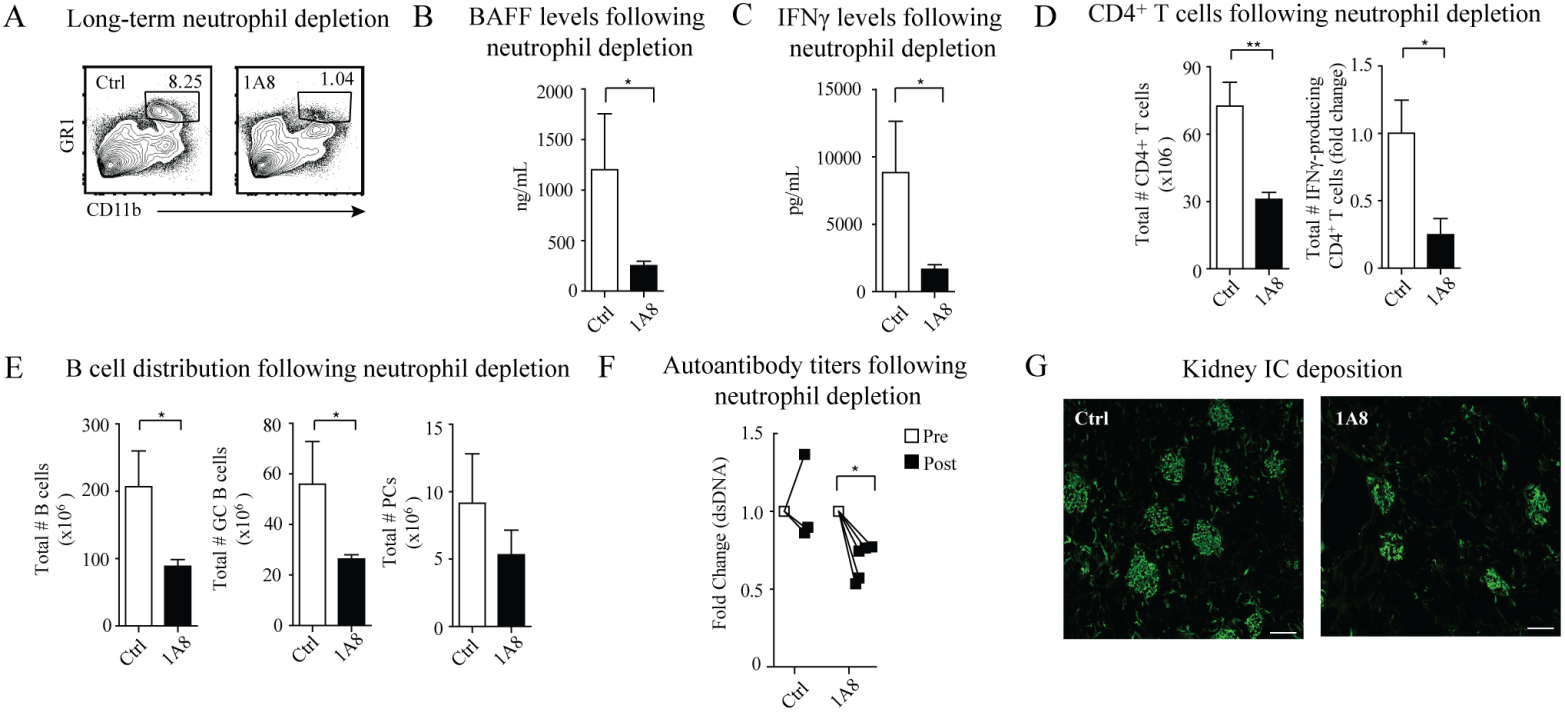
Depletion of neutrophils ameliorates autoimmune disease. B6.Fas^lpr^/J*Tnfrsf17* ^−/−^mice were treated with the 1A8 neutrophil depleting antibody or a control antibody. After 4 weeks, sera, kidney and spleen were analyzed for markers of autoimmune disease. (A) Representative flow cytometry plots showing reduced frequency of neutrophils (CD11b^+^GR1^+^) in spleens after treatment. One plot from 5 mice/group is shown. (B, C) Serum BAFF and IFNγ levels were measured after treatment by ELISA. Combined data from 5 mice/genotype. (D) Total numbers of CD4^+^ T cells and IFNγ-producing CD4^+^ T cells were quantified from spleens of mice after treatment. Combined data from 5 mice/group. (E) Total numbers of B cells, GC B cells (CD19^+^GL7^+^CD138^−^), and PCs (CD19^+/low^CD138^+^) in spleens of mice after treatment were determined by flow cytometry. (F) Serum dsDNA IgG titers of mice before and after treatment were determined by ELISA. Each symbol represents the fold change of autoantibody titers from an individual mouse after treatment. (G) IgG and C3 deposition in kidneys of mice was measured after treatment. One representative image from 5 mice/group is shown. (A–G) One of two independent experiments with similar results is shown. Error bars indicate mean ± SEM. Statistics determined with a Student’s t test (B–E), or a two-way ANOVA using Bonferroni post test (F), and denoted as follows: *p<0.05, **p<0.01.

The above *in* *vitro* findings support the possibility that, like DCs present in spleens of B6.Fas^lpr^/J*Tnfrsf17^−/−^* mice [14], [38], neutrophils may also serve as a key source of BAFF for promoting IFNγ production and humoral autoimmunity *in* *vivo*. To address this possibility, we examined the impact of 1A8 antibody mediated (anti-Ly6G [39]) *in* *vivo* depletion of neutrophils in B6.Fas^lpr^/J*Tnfrsf17^−/−^* mice on BAFF and IFNγ production, germinal center B cell responses, and autoantibody titers after 4 weeks of treatment. Administration of the Ly6G depleting antibody reduced splenic neutrophil frequencies by 80% (Fig. 4A). CD11b^+^ monocytes and dendritic cells expressing intermediate levels of GR1 were unaffected by 1A8 depletion. Neutrophil depletion resulted in a substantial decrease in both serum BAFF and IFNγ levels (Fig. 4B and 4C). We found reduced numbers of CD4^+^ T cells, including IFNγ-producing T cells, in spleens of B6.Fas^lpr^/J*Tnfrsf17^−/−^* mice compared to isotype antibody-treated mice (Fig. 4D). Since reduced levels of BAFF and CD4^+^ T cell help would be expected to affect peripheral B cell responses, we evaluated spleen cellular responses. Mice that were neutrophil depleted had significantly decreased numbers of B cells, germinal center B cells, and plasma cells (Fig. 4E). Moreover, we observed reduced serum anti-dsDNA IgG titers and immune complex deposition in kidneys of mice after neutrophil depletion (Fig. 4F and 4G). These data indicate that neutrophils drive autoimmunity by promoting abnormal BAFF and IFNγ production.

## Discussion

Neutrophils play a key role in the innate host defense to infection [40], [41]. They eliminate pathogens through phagocytosis, the release of antimicrobial products, and the release of neutrophil extracellular traps (NETs) that trap and destroy invading pathogens. In addition, neutrophils have emerged as important regulators of adaptive immune responses to infection by modulating antibody production, dendritic cell activation, and antimicrobial T cell responses. The abnormal accumulation and function of neutrophils are linked with autoimmune diseases such as SLE, yet their role in disease pathogenesis is unclear. Neutrophils derived from SLE patients have impaired phagocytic capacity, increased activation state and, in some patients, a propensity to release NETs [16], [18], [42]. Thus, neutrophils in autoimmunity may contribute to the presentation of autoantigens, leading to autoantibody production. Neutrophils are also a source of cytokines, including BAFF, that are important for B cell development, differentiation, and survival [21], [22], [23], [28]. In lupus-prone mice and SLE patients, increased circulating BAFF levels correlate with increased numbers of peripheral B cells, B cell hyperactivity, and autoantibody production [43], [44]. Thus, neutrophils may also play immunoregulatory roles in autoimmunity in secondary lymphoid organs by producing excess BAFF that promotes survival of autoreactive B cells and the production of autoantibodies. Here we found that the accumulation, activation, and BAFF expression of neutrophils in lupus-prone mice are increased further in the absence of BCMA. We also show that neutrophils from BCMA deficient lupus-prone mice induce CD4^+^ T cell proliferation and IFNγ production in a BAFF-dependent manner more potently compared to neutrophils from BCMA sufficient lupus-prone mice. These findings correlated with higher BAFF and IFNγ serum levels, as well as increased frequencies of IFNγ-producing CD4^+^ T cells, in BCMA deficient lupus-prone mice compared to control animals. Reduced BAFF and IFNγ serum levels, decreased frequencies of IFNγ-producing T cells, GC B cells, and autoantibody production after neutrophil depletion indicated the involvement of neutrophils in these autoimmune traits.

The relevant BAFF receptor(s) through which increased BAFF levels promote lupus is unknown. Given that signals transduced through BR3 on mature B cells and through BCMA on plasma cells support cell survival, it has been thought that the expression of these receptors on B lineage cells is largely responsible for BAFF-mediated B cell hyperactivity and autoantibody production in SLE. However, both *lpr* and *Nba2* mice deficient in BCMA unexpectedly develop an accelerated lupus-like disease that is associated with aberrant BAFF levels, germinal center formation, and CD4^+^ T cell-dependent autoantibody production [14], suggesting an important role for BCMA in the context of murine lupus. Our findings indicate that BCMA critically influences BAFF production by controlling the accumulation and activity of spleen-resident neutrophils. BAFF signaling through BR3 expressed on T cells has been shown to induce proliferation and cytokine production [7], [10], [34], [35]. Thus, apart from excess BAFF directly affecting the activation and survival of autoreactive B cells, BAFF-dependent regulation of CD4^+^ T cell responses may also contribute to B cell hyperactivity and loss of tolerance in autoimmunity.

Although B6.Fas^lpr^/J*Tnfrsf17^−/−^* neutrophils induce CD4^+^ T cell proliferation and IFNγ secretion through BAFF, IL-4 and IL-17 production were driven via an alternative mechanism. These findings are consistent with previous reports demonstrating that neutrophils induce IL-17 via an MHC-dependent mechanism [36], [45]. Importantly, in autoimmune-prone mice, neutrophils localize within the T cell zone *in* *situ*, suggesting our *in* *vitro* findings are biologically relevant. Although the role of type I IFNs in SLE has been well established, the significance and impact of the type II IFNs, has only more recently been appreciated. IFNγ is elevated in the sera of SLE patients [46], [47], [48]. Furthermore, multiple groups have shown the benefits of disrupting the IFNγ signaling pathway in autoimmune disease. Both MRL/*lpr* and NZBWF1 mice deficient in IFNγ or the IFNγ receptor showed significant improvement in disease [49], [50], [51]. Our findings suggest that neutrophils may contribute to increased IFNγ production. Consistent with this notion, depletion of neutrophils *in* *vivo* led to a substantial reduction in serum IFNγ and IFNγ-producing CD4^+^ T cells, as well as an overall improvement in disease severity.

BCMA expression was not detected in neutrophils. This finding indicates that BCMA deficiency in neutrophils themselves is not involved in the differences of neutrophil accumulation and activation between B6.Fas^lpr^/J and B6.Fas^lpr^/J*Tnfrsf17^−/−^* animals. We propose that the overall inflammatory environment in B6.Fas^lpr^/J*Tnfrsf17^−/−^* mice affects neutrophil accumulation compared to B6.Fas^lpr^/J mice [14]. How neutrophils are recruited to secondary lymphoid organs is unknown; however, IFNγ has been shown to mediate neutrophil recruitment to the lung following tissue injury or infection [52], [53]. Thus, further work will be necessary to determine whether a feedback loop exists between BAFF-producing neutrophils and IFNγ-producing CD4^+^ T cells in the recruitment of neutrophils in autoimmune disease. Taken together, our findings demonstrate a role for neutrophils in the pathogenesis of murine lupus.
